# Rachel Challice's Vow

**Published:** 1888-03-10

**Authors:** Lina Nevill

**Affiliations:** Author of "A Future on Trust," "Juno," &c.


### Rachel Challice's Vow.

By Lina Nevill, Author of "A Future on Trust," "Juno," &c.

### Chapter XII.—The Old Home.

"Endure—and there shall dawn within thy breast,
Eternal rest."—*Young.*

It was a lovely late September evening when the Endsleighs, Rachel with them, arrived at Bedd-gelert. The air was chilly, but the sun was still shining on the mountain-sides; and the hills and ranges stood out sharp and clear—great, rough, mighty masses against the rose-flushed evening sky. Hebog loomed purple and dark above Bedd-gelert village, his great form casting a mighty shadow over the narrow valley. Arran, on the other side of the road, shot up her peak into the sky in imitation of greater Snowdon, whose queenly summit was crowned with a glittering sunny diadem, marking her right to sovereignty, and singling her out from her crowd of subjects.

Away far in the distance, beyond Dinas and Gwynaut, and the gloomy entrance to Llanberis, by Capel Curig rose beautiful Siabod, her graceful form bathed in a sea of rosy glory, poured over her barren sides by the rays of the dying sun.

Even the cheerless road from Ryhd-ddu to Bedd-gelert looked less dreary than usual. The moor had begged a gleam of brightness from the sun; and decking itself in the robe of borrowed golden lustre, it spread out in red and brown richness of colour, with foaming Colwyn singing its wild song, as it rushed and tumbled along its narrow rocky bed.

"I like it all better than anything I have ever seen," cried Nell ecstatically; "you never told me we were coming to anything half so lovely," she added, addressing Rachel, who sat opposite to her. "I should like to live here always—would not you, mother?"

Mrs. Endsleigh shivered, partly at the prospect called up by her daughter's words, partly on account of the chilly evening air. It felt cold to her after the warm railway carriage; and the breeze blowing straight from the sea and the bare hillsides was sharp and bracing.

"No, my dear," she replied decidedly. "I should dislike living here for any length of time extremely. However, if Charley and you agree in adoring the place, there is no reason whatever why you should not make it your permanent abode. Only do not expect me to come and visit you," she added, with a little laugh. "If Charley dislikes the notion of a mother-in-law meddling in all his affairs, let him settle here, and he will have nothing to fear from me on that score!"

"You are a regular town mouse, mother," said Nell disdainfully. "Nurse Challice, don't you like all this wildness and beauty far better than London or Brighton, or any of those places where people go to see each other instead of the country? I am sure you must. I cannot believe you have such bad taste as to prefer a town to this beautiful wilderness."

Rachel roused herself with a start.

"I, Miss Endsleigh? Oh, I think I like London best. You know this is not new to me; I have seen it all before."

She had been gazing with sad, vacant eyes at the well-known scene around her. Every tree, almost every stone by the roadside, looked familiar.

She could hardly believe that more than three years had passed since she left it all; everything looked so little changed. And yet, before coming, when she had glanced back on the time, it had seemed an eternity—a past so far removed as to appear like a vivid dream, or a story read out of a book and then half forgotten.

Now the past rushed back upon her so forcibly as to become once again a real living present. The intervening years were forgotten. Once again she was Rachel Challice—the despised, hated grandchild, the disdained devoted sister.

The carriage was driving by her old home at the moment, past the house where she and Esther had lived for so long, and where her grandmother had died.

At first sight of it Rachel felt as though she *must* call out to the coachman and tell him to stop. She almost fancied she would see Esther standing watching for her at the gate, and hear her fretful, spoilt-child voice complaining that she had been left so long alone: where had Rachel been all this time? why had she gone away and given her all the work to do?

As they drove close to the house the vision was partly dispelled. Mrs. Howells was evidently still in full possession, and apparently in the same state of house-cleaning as on the day Rachel had left.

Tables and chairs were piled in confusion outside the door. A carpet hung out over one of the open upper windows. Mrs. Howells, in the identical black bonnet she had worn three years ago, was busily dusting her mats by dashing them vigorously against the garden wall, an occupation from which she desisted for a moment as the carriage drove by; and as she peered curiously into the vehicle her sharp eyes caught sight of Rachel.

She recognised her in a minute. Mrs. Howells was the kind of woman you would set to find a needle in a bundle of hay, were you particularly anxious to find it, so certain would you be that, if it was there at all, she would discover the hidden treasure.

She tilted back her bonnet a little further off her head, stared fixedly into the girl's face, and then gave her a little nod of recognition.

"That woman knows you, Nurse," said Nell quickly; her eyes were nearly as sharp as those of Mrs. Howell.

"Yes. I knew her a little long ago. She has a good memory," replied Rachel, with as careless a manner as she could assume.

She tried not to mind; she told herself over and over again impatiently that she was foolish to do so; that this was but the beginning of what must certainly daily happen.

But she was vexed at the encounter, and at the instantaneous recognition. It showed that there was no chance of people having forgotten her. Mrs. Howells was an inveterate gossip. Rachel knew as well as if she had been present that the woman would post off to Bedd-gelert or Aberelwyn the moment her cleanings were accomplished, and tell every man, woman, and child whom she met that Rachel Challice had come back, that she had seen her that very afternoon driving in a grand carriage from Ryhd-ddu toward Bedd-gelert, and that she (Mrs. Howells) had heard that the said carriage belonged to some fine London people who had come down to stay at the hotel for the autumn.

The carriage drove over the bridge and for a short distance up the road leading to Pont Aberglaslyn and Portmadoc.

The tall fir-clad mountains, the opposite side, loomed black and sombre in the gathering dusk. The sun had set behind Siabod. Snowdon's crest was veiled in mist, and the murmur of the rippling Glasslyn came soothingly to their ears in place of the wild, rushing Colwyn. A white mist was rising in the valley, and meeting the clouds that hovered over the heads of the mountains. The sheep-bells were silent; the bats whirled blindly in the still air, and the hush of night fell over the wild mountain land as Rachel Challice once more came home.

### Chapter XIII.—Rachel's First Visit.

"But who could bear that gloomy blank,
Where joy was lost, as well as pain?
Quickly of Memory's fount I drank,
And brought the past all back again."
—*Moore.*

All the next day Rachel stayed indoors.

Nobody remarked it; and she pretended she was too busy arranging her own and Miss Endsleigh's belongings to think of anything else.

Nell was too tired in the morning from the effects of her journey to care about taking Rachel out with her; and in the afternoon she drove for a short time with Sir Charles, returning charmed with the beauty of the country and full of plans for future excursions—plans which Mrs. Endsleigh listened to with a patient, forbearing smile.

With all the contrariety of human nature Rachel in a day or two found herself as eagerly anxious to see Esther and her husband as before she had dreaded and shrunk from the meeting.

After all, her sister was the one near relation she had in the world; the ties of blood are wonderfully strong and enduring, even though they may lie dormant for a time. In her constrained, peculiar fashion she loved Esther as devotedly as ever. It was the meeting with David that she really dreaded. She knew she exaggerated in her own mind the pain such an encounter would cause; that the reality would be far less than the idea; that when all was over she would laugh at herself for her foolish fancies. And yet she could not conquer the feeling.

On the evening of the second day, just before Nell went to dress for dinner, Rachel asked leave to go out for an hour or two by herself.

As soon as she left the hotel Nurse Challice took the direct road to Aber-elwyn.

Esther, before her marriage, had told Rachel that she and David were going to live with his father, David having at that time considered such an arrangement a foregone conclusion. Therefore, as soon as she reached the village she bent her steps in the direction of Elias Stephens' house.

The blind was down, but a bright light gleamed in one of the lower windows.

With a beating heart Rachel unlatched the garden-gate, and walked up the path through the trim, well-kept garden.

Her cheeks burned both from excitement and with her rapid walk in the fresh air. Her eyes were large and bright. She had no idea how beautiful she looked at that moment; how unlike the pale, cold, expressionless girl she had been when she left Ryhd-ddu.

She longed for, yet dreaded, the meeting in store for her. Her heart leaped up at the prospect of seeing her darling Esther a happy wife—perhaps even by this time a loving mother. She wondered if Esther would be glad to see her; if absence had made the heart grow fonder, or only rendered it more indifferent and careless.

With trembling, eager fingers she knocked at the door, and listened anxiously for a response.

Footsteps came slowly along the narrow flagged passage. Surely that could not be Esther's brisk young tread! She must have changed marvellously in the three years if these slow heavy steps belonged to her.

A bright light flashed in Rachel's eyes. A hard voice asked, —"Who is there?"

This was not Esther—not her pretty gay young sister. It must be some servant. It was certainly extravagant to keep a maid in the house; but so like Esther's ways—anything rather than do the work herself. And no doubt both her husband and father-in-law combined to spoil her; and willingly paid extra rather than see her bright spirits dashed by the burthen and toil of housekeeping.

"I want to see Mrs. Stephens," she said pleasantly.

"I don't know who you mean," responded the hard gruff voice, as the woman, standing inside the door, took stock of Rachel, looking her over by the light of the lamp she carried in her hand.

"The old man is in," she went on. "I daresay you can see him if you choose."

"That will do just as well. I will wait with him till the others come in."

Evidently Esther's nature had not changed much. Her old love of gadding about must still be strong within her. But it was very harmless so long as her husband went too; and the woman had distinctly said that the old man was the only one at home. Now she remembered it was Portmadoc market-day. Very likely Esther and David had gone to the town and were late in returning. No doubt that was the reason for their absence.

The servant led the way silently along the passage, Rachel following.

Then she paused, threw open the door of the kitchen, and saying briefly, "A young woman to see you," pushed Rachel inside the door, and her slow, heavy footsteps might be heard retreating to some back region of the house.

There was no light in the kitchen but that of the blazing fire, which threw a flickering ruddy glow over the room, and showed Rachel the figure of the old man sitting in his wooden arm-chair, dozing, before the fire.

"Who is that, Martha? Who did you say wanted me?"

"It is I, Mr. Stephens," said Rachel, advancing out of the shadow, so that the blaze from the fire fell full upon her, and showed her face to the old man.

"Hey! You! So you are here after all. That old gossip Mrs. Howells came to the mill this afternoon to buy some flour—I don't believe she wanted it, she only came to use her tongue—and tried to tell me some long cock-and-bull story about having seen you driving in a grand carriage, and staying up at the Bedd-gelert Hotel, with a barrow-nite and his family, and I don't know what rubbish besides."

"And you would not believe her?" said Rachel, smiling.

"Believe her!" repeated Elias scornfully. "'My good woman,' says I—though I don't believe as how she is a good woman—'you say you've come for some flour; take it, and go as quick as you can. I'll trouble you for nothing but the money in return. You can keep the interest of your talk for them as likes sixty per cent. extra. I'm satisfied with my just due, and wishes for nothing beyond.' She didn't trouble the mill long with her company after that!" concluded Elias, with a complacent chuckle.

"Poor Mrs. Howells!" remarked Rachel. "Still, she was right after all in most of her story. She did see me in the carriage, and I am staying at the Bedd-gelert Hotel. I came there from London the day before yesterday."

"Hey! The old woman was right! Why didn't you say so at once then, instead of deceiving me all this time?" returned Elias testily, rather crestfallen to find that Mrs. Howells was more correct in her information than he had believed. "What are you doing down here, pray? And why have you come to see me?"

"I have come down with the family of a young lady whom I have been nursing. I am a trained nurse now."

"That is a meritorious calling," observed Elias in a patronising tone. "And it seems to agree with you. I never saw you looking better."

"I have not come purposely to see you," went on Rachel calmly. "I want to see my sister. How soon will she be in?"

"Your sister!" he repeated in a slow, astonished voice, gazing at her inquiringly through his heavy silver-rimmed spectacles. "If it's her you've come to see, Rachel Challice, all I can say is you've come to the wrong house."

"I don't understand you," she said in a puzzled tone. "Please explain quickly what you mean. I have not very much time, and I want to see Esther particularly before I go back to Bedd-gelert."

"If that's your object, it is a pity you took the trouble to come out to Aber-elwyn," returned Elias tartly. "You might have saved yourself the walk. It's no business of mine, but, as far as I know, your sister is in Bedd-gelert village this very minute."

"But how soon will she be back? I want so much to see her. I may miss her if I go to meet them."

"I don't think she is particularly likely to be out; leastways, she wouldn't be if she was doing her duty. David is ill, so he sent word a week ago to me, and he hasn't been near the mill since; and a wife's place, as I consider, is by the side of her sick husband."

"Then do you mean that she and David do not live here any longer?" asked Rachel, light at length beginning to dawn upon her perplexity. "You know," she went on hurriedly, "I have been so long away, and I—we—that is, there has been so little correspondence, I really know nothing about them or their present way of living."

"Nor I either," responded Elias drily. "You couldn't come to a worse place than this to find out about them. We seem quits, you and I, in leaving the young couple to themselves. I never liked your sister. I was against the match from the first, and David knew all along what my feelings on the subject were. They came here on the wedding morning expecting to be taken in. I says, 'As you've made your bed so you must lie on it.' And I haven't seen your sister's silly, dolly face inside my door from that day. No, nor I never wish to, neither."

"You are a cruel, heartless old man!" cried Rachel indignantly. Though the truth of Elias' remark as to her having been equally indifferent to and careless of her sister, struck her as being justly true.

"Highty-tighty! Are these London manners?" asked the old man coolly, and without any show of offence. "I suppose I've a right to choose my own visitors in my own house. Your sister invited herself, and I showed her the way to the door, along with her young idiot of a husband. And I'd do it again if they was to try the same game on. And you may tell them so from me."

"Where do they live now?" asked Rachel coldly. "I will go and see them at once, if you will tell me the house."

"I don't know myself, Rachel Challice; I never had the curiosity to inquire. I know it is somewhere in Bedd-gelert, for I've heard David mention what he has seen in the walk when he comes in the morning to the mill. Beyond that I can't say; but no doubt Martha knows. Trust a woman to find out all about her neighbours' affairs."

He went to the door and called the gaunt handmaiden, who, as he suspected, was able to give the desired infor mation. With a curt "Good-night" Rachel left the house and set out on her return journey to Bedd-gelert.

(*To be continued.*)

